# MicroRNA‐140‐5p inhibitor attenuates memory impairment induced by amyloid‐ß oligomer in vivo possibly through Pin1 regulation

**DOI:** 10.1111/cns.13980

**Published:** 2022-10-02

**Authors:** Pariya Khodabakhsh, Maryam Bazrgar, Fatemeh Mohagheghi, Siavash Parvardeh, Abolhassan Ahmadiani

**Affiliations:** ^1^ Department of Pharmacology, School of Medicine Shahid Beheshti University of Medical Sciences Tehran Iran; ^2^ Neuroscience Research Center Shahid Beheshti University of Medical Science Tehran Iran; ^3^ Institute of Experimental Hematology, Center for Translational Cancer Research (TranslaTUM), School of Medicine Technical University of Munich Munich Germany

**Keywords:** ADAM10, Alzheimer's disease, amyloid‐β oligomer, insulin signaling, miR‐140‐5p, Pin1

## Abstract

**Aims:**

The peptidyl‐prolyl *cis/trans* isomerase, Pin1, has a protective role in age‐related neurodegeneration by targeting different phosphorylation sites of tau and the key proteins required to produce Amyloid‐β, which are the well‐known molecular signatures of Alzheimer's disease (AD) neuropathology. The direct interaction of miR‐140‐5p with Pin1 mRNA and its inhibitory role in protein translation has been identified. The main purpose of this study was to investigate the role of miRNA‐140‐5p inhibition in promoting Pin1 expression and the therapeutic potential of the AntimiR‐140‐5p in the Aß oligomer (AßO)‐induced AD rat model.

**Methods:**

Spatial learning and memory were assessed in the Morris water maze. RT‐PCR, western blot, and histological assays were performed on hippocampal samples at various time points after treatments. miRNA‐140‐5p inhibition enhanced Pin1 and ADAM10 mRNA expressions but has little effect on Pin1 protein level.

**Results:**

The miRNA‐140‐5p inhibitor markedly ameliorated spatial learning and memory deficits induced by AßO, and concomitantly suppressed the mRNA expression of inflammatory mediators TNFα and IL‐1β, and phosphorylation of tau at three key sites (thr231, ser396, and ser404) as well as increased phosphorylated Ser473‐Akt.

**Conclusion:**

According to our results, Antimir‐140‐mediated improvement of AβO‐induced neuronal injury and memory impairment in rats may provide an appropriate rationale for evaluating miR‐140‐5p inhibitors as a promising agent for the treatment of AD.

## Graphical abstract

miRNA‐140‐5p inhibitor ameliorated cognitive deficits induced by AßO. Further, miR‐140‐5p downregulation was associated with suppressing neuroinflammation and abnormal tau phosphorylation. This study exhibits a crucial role for miR‐140‐5p in the key events of oligomeric amyloid‐beta induced neurotoxicity, which may serve as a potential therapeutic target for AD.

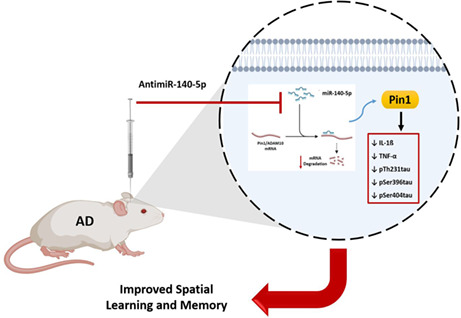

## 1 INTRODUCTION

Alzheimer's disease (AD), as the most common type of dementia in aged subjects, is a progressive neurodegenerative disorder defined clinically by irreversible memory loss and severe cognitive impairment.^1^ Extracellular amyloid‐β (Aβ) plaques and intracellular accumulation of neurofibrillary tangles (NFTs) composed of hyperphosphorylated tau (ptau) are the well‐known molecular signatures of AD. Burgeoning evidence implicates the notion of Aβ–tau synergy in AD neuropathology and therapeutic strategies targeting both pathological proteins may be more beneficial.^2^

Pin1 is a conserved peptidyl‐prolyl cis‐trans isomerase (PPIase) that interconverts cis and trans isomers of proline following binding to phosphorylated proline‐directed serine or threonine residues.^3^ This site‐specific conformational change mediated by Pin1 on its substrate results in its implication in several neurophysiological processes including neurodevelopment, neuronal function, synaptic plasticity, and apoptosis.^4^ Data from numerous studies suggest that Pin1 deregulation plays an integral role in age‐related and neurodegenerative diseases. In the context of AD, Pin1 has been found to inhibit age‐associated neurodegeneration by inducing ptau dephosphorylation and restoring its function through catalyzing the pathogenic *cis* to a physiologic *trans* isomer of Thr231ptau.^5^ Another notion of the pivotal role of Pin1 in the AD pathology process is its stimulatory effects on the non‐amyloidogenic processing of amyloid‐beta precursor protein (APP) by inhibiting glycogen synthase kinase 3 beta (GSK3β) activity and consequently reducing Aβ production.^6^

A growing body of evidence suggests that impaired insulin signaling and insulin resistance at the central level are the main early events in the development of AD.^7^, ^8^ Pin1 has been proposed to positively modulate insulin signaling particularly through regulating the active conformation of Akt protein, an index of insulin sensitivity, and the aforementioned effects on GSK3β kinase activity.^9^ Interestingly, it has been reported that the gene expression of Pin1 in the postmortem human hippocampus is decreased in AD patients in comparison to age‐matched controls.^5^ Therefore, finding a therapeutic strategy to enhance the Pin1 expression or activity will likely afford valuable effects in AD.

Micro(mi)RNAs, are the endogenous, small, non‐coding RNA molecules, that post‐transcriptionally regulate the expression of messenger (m)RNAs. The mature miRNA directs translational attenuation through binding to an mRNA target site for its degradation or translational suppression.^10^ Based on the literature data, miR‐140‐5p contributes noteworthily in regulating the Pin1 mRNA expression. In a study by Yan et al., through bioinformatics analysis, miRNA binding, and functional assays, it has been confirmed that miR‐140‐5p suppresses Pin1 translation via the direct interaction with the 3′ untranslated region (3′UTR) of the target mRNA.^11^

Over the last few years, miR‐140‐5p has attracted interest as a diagnostic and therapeutic target for several diseases including neurodegeneration and oncogenesis. The association between miR‐140‐5p downregulation and various kinds of tumor tissues and cell lines, and the antitumor activity of the miRNA through targeting certain oncogenic genes have been widely documented.^12^, ^13^, ^14^ On the other side, Xing et al. showed that miR‐140‐5p can attenuate cell proliferation and promote apoptosis and thus its inhibition may have the potential to improve hypoxia‐induced cell injury.^15^ The complicated contribution of miR‐140‐5p in several neurological diseases, such as stroke,^16^ multiple sclerosis,^17^ autism spectrum disorder,^18^ AD,^19^ and Parkinson's disease,^20^ has been already reported. miR‐140‐5 has been shown to importantly contribute to alterations in oxidative stress and apoptotic cascades in brain.^21^ A study on the epigenetic changes in stroke patients revealed that plasma levels of miR‐140‐5p significantly increase in the late‐onset post‐stroke depression patients, and thus it can consider as a novel biomarker to early predict the disease.^16^ The predictive role of miR‐140‐5p has been also indicated for age‐related cognitive decline.^22^ However, in a study by Guan et al., an inverse relationship was suggested between the expression of miR‐140‐5p and multiple sclerosis severity.^17^ Interestingly, there is evidence that the upregulation of miRNA‐140‐5p has been identified in the post‐mortem hippocampus of AD patients. Also, it has been indicated that the increased levels of miRNA‐140‐5p can be detected in neuronal SHSY5Y cells following Aβ toxicity.^19^ Liang et al. recently reported that silencing of miR‐140 in brain tissues of AD modeled rats was associated with suppressing mitochondrial dysfunction, and improving cell autophagy.^23^ In the present study, we aimed to assess the therapeutic potential of miR‐140‐5p inhibitor and its possible mechanisms through Pin1 targeting in a rat model of AD.

## 2 MATERIALS AND METHODS

### 2.1 Animals

A total of 64 adult male Wistar rats with an average age of 12 weeks weighing approximately 250–280 g were procured from the breeding colony of Neuroscience Research Center, Shahid Beheshti University of Medical Sciences. The rats were held four per cage under standard animal room conditions at 21 ± 2°C and a 12 h light/dark cycle with free access to food and water ad libitum. In the present study, the rats were sacrificed by CO_2_ inhalation. All experimental protocols involving rats were approved by the Ethical Committee of Neuroscience Research Center, Shahid Beheshti University of Medical Sciences (ethics approval code: IR.SBMU.MSP.REC.1397.530) in compliance with the standards of the European Communities Council Directive (86/609/EEC). All efforts were made to reduce animal suffering and the number of rats needed for the study.

### 2.2 Materials

Anti‐miR‐140‐5p (AM‐140) was synthesized by Metabion (Martinsried, Germany). AM‐140 contained 2’‐O‐methyl modifications (5′‐(2’OMe‐C) *(2’OMe‐U) *a* cca uag ggu aaa acc *(2’OMe‐A)* (2’OMe‐C)*(2’OMe‐U) *(2’OMe‐G)‐3′). Aβ (1–42) (CAS number: ab120301) were purchased from Abcam. Oligomeric Aβ1–42 was prepared as originally established by Klein et al.^24^ with a slight modification. The lyophilized synthetic human Aβ1–42 peptides were dissolved in 1,1,1,3,3,3‐hexafluoro‐2‐propanol (Sigma‐Aldrich, 105,228) and then evaporated overnight at room temperature in aliquots. The resulting Aβ peptide film was stored at −80°C. 24 h before use, the films were resuspended in anhydrous dimethyl sulfoxide (DMSO) to 5 mM, and then diluted in phosphate‐buffered saline (PBS; pH 7.4) to a concentration of 100 μM and stored at 4°C.

### 2.3 Surgery

**FIGURE 1:**
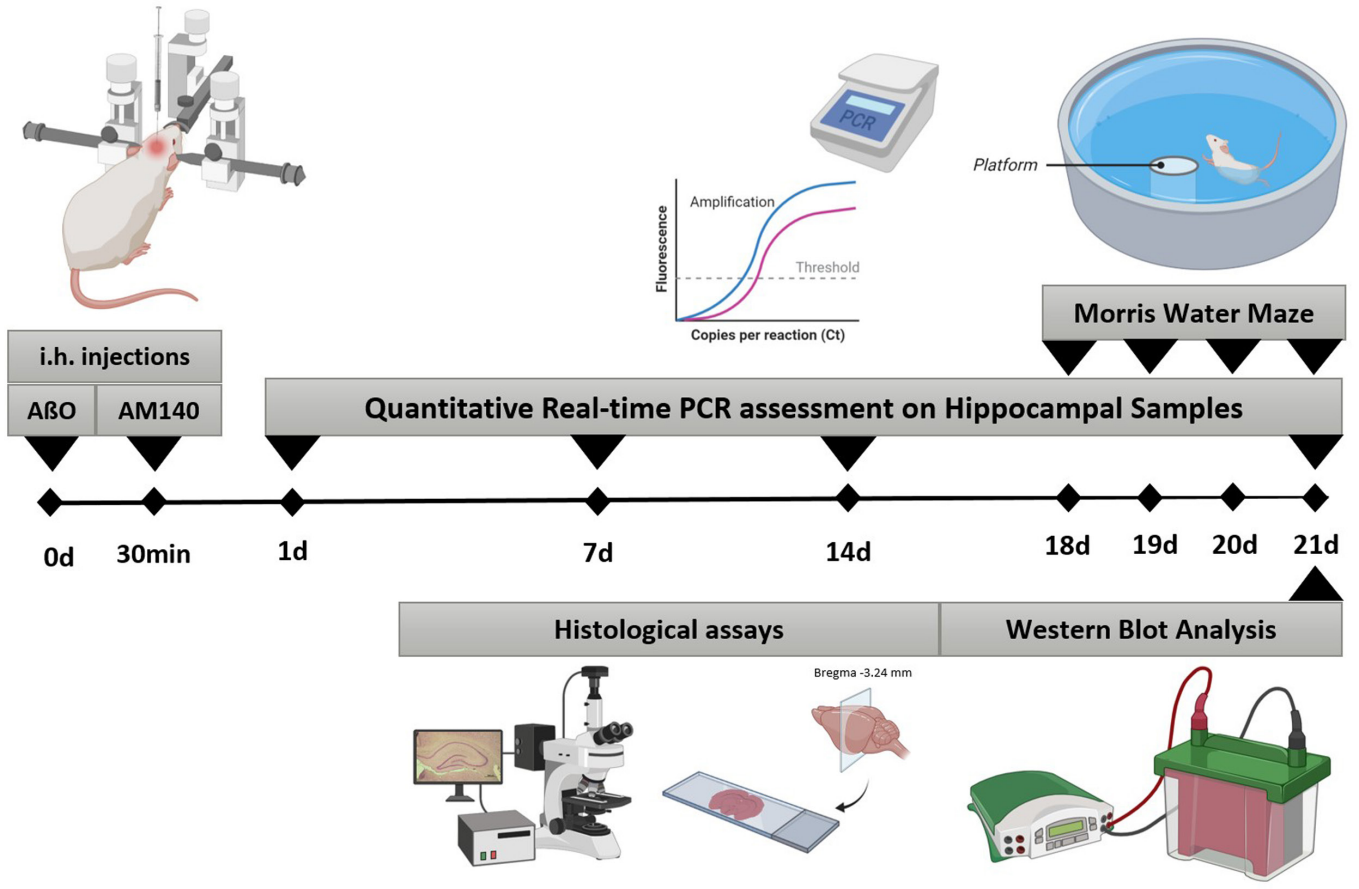
Schematic representation of the experimental schedule. Oligomeric Aβ1–42 (Aβo) and anti‐miR‐140‐5p (AM) were bilaterally injected in the rat hippocampus. Learning and memory abilities were evaluated using the Morris water maze tests on days 18–21. Brain and hippocampus samples were collected from each group at different time points (24 h, 7, 14, and 21 days) after the stereotactic injections in order to molecular and histological assays (created by Biorender.com)

The rats were randomly distributed into four equal groups with 16 rats in each one as follows: control group received AβO vehicle (containing phosphate‐buffered saline [PBS] and DMSO) and AM‐140 vehicle, AβO group received oligomeric Aβ and AM‐140 vehicle; AM group received Aβo vehicle and AM‐140 vehicle; AM+AβO group received oligomeric Aβ in combination with AM‐140. Stereotactic (Stoelting) surgery was carried out for the bilaterally intrahippocampal injections under anesthesia using a mixture of ketamine and xylazine (100 and 10 mg/kg, respectively). Oligomeric Aβ 1–42 (2.5 μg/μl, 3 μl in each side) and AM‐140 (10 pmol/μl, 1 μl in each side) were injected into the CA1 region of the rat hippocampus (AP: −3.84 mm, ML: ±2.2 mm, DV: −3.3 mm according to rat brain atlas,^25^ at a flow rate of 0.5 μl/min using an injection needle (27 G) connected to a 25 μl Hamilton syringe (Hamilton). AM‐140 or its vehicle was injected sequentially within 30 min after AβO or its vehicle administration using the same injection parameters. The experimental procedure is schematically presented in Figure 1.

### 2.4 Behavioral assessment

Spatial learning and memory of rats were assessed 18 days after stereotaxic surgery according to a modification of the procedure previously established by Morris.^26^ In short, the water maze composes of a standard black circular tank (150 cm diameter, 70 cm height, 45 cm deep) filled with water at 22 ± 1°C to a depth of 30 cm. The tank was divided into four equal quadrants including northeast (NE), northwest (NW), southeast (SE), and southwest (SW) by EthoVision tracking system software (Noldus Information Technology, Wageningen, The Netherlands).^27^ A dark hidden platform was placed in a fixed position in the center of the NE quadrant and submerged below the surface of the water (approximately 2 cm). Several pictures as extra‐maze visual cues were hung around the tank.

This test includes a place navigation (Learning) phase and a probe phase. The place navigation test (training) was carried out as four trials daily for three consecutive days to induce learning starting on day 18 following surgery. In each trial, the animal was inserted into the water facing the wall of the pool at one of the quadrants, allowed to swim, and expected to find the hidden platform within the 60 s. An animal that failed to find the platform was manually guided and let stay there for 20 s for reinforcement. On the 4th day of the experiment, 24 h after the last acquisition trial, the probe phase was conducted without the platform and each rat was left to swim within 60 s. The rats were expected to spend the longest time in the quadrant containing the platform (target quadrant). All behavioral parameters, including traveled distance, escape latency (time to find the platform), and swimming speed in the learning phase, as well as the time spent in the target quadrant, the number of crossings over the platform in the probe phase, were recorded by a video camera and analyzed through EthoVision program.

### 2.5 Tissue collection and preservation

Brain and hippocampus samples were collected and preserved differently based on the following analysis methods. Three rats from each group were sacrificed at different time points (24 h, 7, 14, 21 days) after the stereotactic injections, snap‐frozen, and kept at −80°C to be later used for molecular and biochemical assessments (Figure 1). In order to histological analysis, rats (*n* = 3) from each group were deeply anesthetized and perfused transcardially using ice‐cold PBS (10 mM, pH. 7.4), followed by 4% paraformaldehyde (PFA) in PBS. Then, the whole brains were isolated and post‐fixed in PFA for at least 72 h at 4 °C. Following the gradient dehydration, the tissues were embedded in paraffin wax at 59 °C and sectioned to a thickness of 5–7 μm.

### 2.6 RNA extraction and RT‐PCR

The real‐time polymerase chain reaction (RT‐PCR) method was employed to quantify the expression levels of miR‐140 and mRNAs encoding Pin1, ADAM10, IL‐1ß, and TNFα in hippocampus samples collected 24 h, 7, 14, and 21 days post‐surgery. Initially, the total RNA was extracted from the tissues by the means of a GeneAll Hybrid‐R RNA purification kit (GeneAll) based on the instruction included in the kit. The concentration of the purified RNAs was measured by Nanodrop at 260 nm (Thermo Fisher Scientific). The expression analysis of miR‐140 was quantified using stemloop RT‐qPCR. Complementary deoxyribonucleic acid (cDNA) and PCR products were synthesized by the SYBR green MicroRNA kits (AnaCell) for rno‐miR‐140 in accordance with the manufacturer's instructions. A primer set for the U6 gene was also included in the kit as the endogenous control. The reaction protocol contained the following steps: heating for 15 min at 95°C, followed by 40 cycles of amplification (15 s at 95°C and 1 min at 60°C). After the last amplification cycle, melting analysis was performed by one cycle of heating to 95°C.

To analyze the mRNA expressions, a cDNA synthesis kit with OligodT primers (Solis BioDyne, Tartu, Estonia) was employed for RNA reverse transcription to cDNA in a final volume of 20 μl. Transcript levels of Pin1, ADAM10, IL‐1ß, and TNF‐α were assessed by an ABI 7500 StepOnePlus™ Real‐Time PCR System (Applied Biosystems) with RealQ Plus 2x Master Mix Green, High ROX TM (Ampliqon, Denmark) and primers (Metabion, Martinsried) listed in Table 1, under the following thermocycling parameters: 95°C, 10 min, succeeded by 40 cycles of 95°C (15 s), 60°C (30 s, and 72°C (30 s). β‐actin mRNA was utilized as an internal control to measure the relative expressions of target genes). The fold change of gene expression was calculated using the 2^−ΔΔCt^ method.^28^

**TABLE 1:** Primers Used in Real‐Time RT‐PCR Analysis

Gene	Forward primer (5′–3′)	Reverse primer (5′–3′)	Amplicon length
PIN1	TGGGAGAAGCGTATGAGTCG	CGAGATTGGCTGTGCTTCAC	179
ADAM10	GGTTTCATCCAGACTCGGGGT	TGAAACGGCAGGATTCGGTCT	80
IL‐1ß	TGCCACCTTTTGACAGTGATG	TGATGTGCTGCTGCGAGATT	138
TNF‐α	ACTGAACTTCGGGGTGATCG	CGCTTGGTGGTTTGCTACG	154
ß‐Actin	GCAGGAGTACGATGAGTCCG	ACGCAGCTCAGTAACAGTCC	74

### 2.7 Western blot analysis

Western blot analysis was performed based on previous protocols.^29^ Briefly, samples were lysed individually in the presence of five volumes of lysis buffer. Then, the lysates were clarified by centrifugation for 20 min at 12000*g* and 4°C, and the supernatants were collected. The total protein concentration was determined through Bradford assay against BSA as standard. The lysates were then fractioned by 12.5% sodium dodecyl sulfate‐polyacrylamide gel electrophoresis (SDS‐PAGE), and blotted onto polyvinylidene difluoride (PVDF) membranes with a pore size of 0.45 mm (Millipore). 5% non‐fat dry milk (Sigma, USA) dissolved in Tris‐buffered saline with 0.1% Tween® 80 detergent (TBST) was used as membrane blocking solution. After 75 min blocking, the blots were probed with primary antibodies by overnight incubation at 4°C with antibodies against Pin1 (dilution 1:200, cat. no. ab12107; Abcam), p‐Tau (Thr231) (1:1000, cat. no. SAB4504563; Sigma‐Aldrich), p‐Tau (Ser396) (1:1000, cat. no. 44‐752G; Invitrogen), p‐Tau (Ser404) (1:1000, cat. no. sc‐7985‐R; Invitrogen), p‐GSK3ß(Ser9) (1:500, cat. no. PA1‐4688; Invitrogen), p‐Akt1/2/3 (Ser 473) (1:150, cat. no. sc‐7985‐R; Santa Cruz), and β‐actin (1:750, Cell Signaling, USA, #4970). Subsequently, the membranes were incubated with secondary horseradish peroxidase (HRP)‐conjugated goat anti‐rabbit antibody (1: 10,000; ab6721, Abcam) for 2 h at room temperature. The enhanced chemiluminescence reagent (ECL) (Bio‐Rad) was used to visualized protein bands on x‐ray films. The relative protein expression levels, normalized to β‐actin, were quantified using ImageJ program (National Institutes of Health).

### 2.8 Histochemical staining

Coronal sections were stained as previously described.^30^, ^31^ Briefly, following deparaffinizing and hydrating with xylene and descending graded ethanol, the sections were incubated in 0.1% cresyl Violet solution (Nissl Staining, Sigma‐Aldrich), for 6 min at 58°C. Subsequently, they were dehydrated using increasing concentrations of ethanol, cleared by xylene, and cover‐slipped. Five to seven cresyl violet‐stained sections of each animal at a level of 2.7–3.7 mm posterior to the bregma, were selected for evaluation by the light microscope (Nikon). The CA1, CA2, CA3, and DG regions were assessed and taken photos at fields of 40 and 200× magnification. The cells with a round shape, a well‐defined nucleolus, and typical Nissl bodies in the cytoplasm were known as normal and surviving neurons.^32^ Cells with abnormal morphology, massive shrinkage, dense cytoplasm, and dark nucleus were considered dark neurons.^33^

### 2.9 Statistical analysis

Statistical analyses were completed using GraphPad Prism software version 6 (GraphPad Software, Inc.,), and all data are presented as mean ± standard error of the mean (SEM). Either one‐way analysis of variance (ANOVA) or two‐way repetitive measure ANOVA followed by Tukey's multiple comparisons post‐hoc tests were conducted where appropriate. Shapiro–Wilk normality test indicated that the data have normal distribution; therefore, comparisons were done using parametric tests. Data followed by *p* < 0.05 were considered to indicate statistical significance.

## 3 RESULTS

In the present study, we used AM‐140 contained 2’‐O‐methyl modifications. Based on previous investigations, anti‐miRNA oligonucleotides with complementary sequences of the target miRNA bind miRNAs, form a duplex and inhibit miRNA functions in living cells.^34^ The chemical modification was used to enhance affinity to RNA targets and nuclease resistance.^35^, ^36^

### 3.1 mir‐140‐5p inhibitor restores spatial learning and memory impairment induced by AßO

**FIGURE 2:**
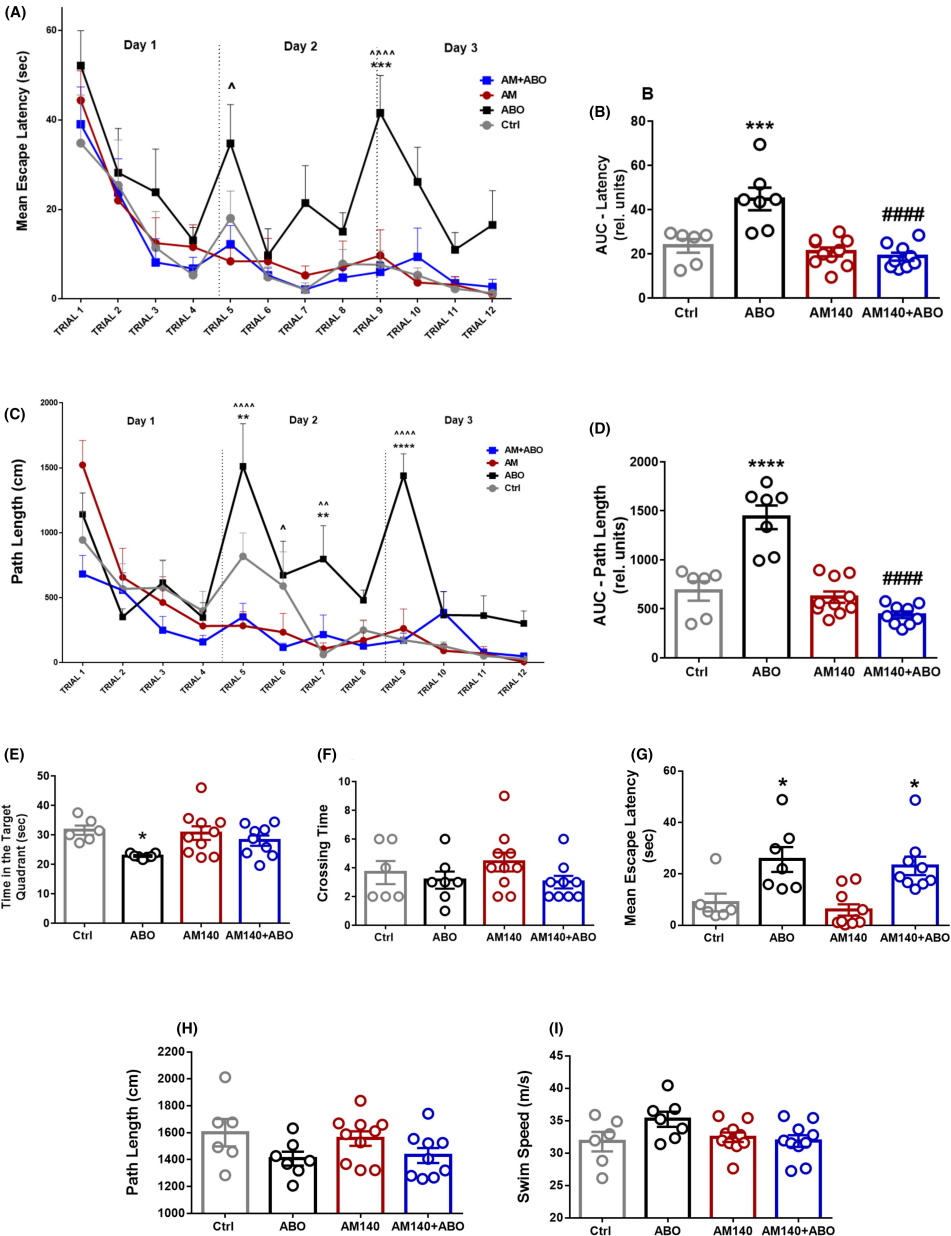
Effect of AM‐140 on spatial learning and memory impairment in the hippocampus‐dependent memory task Morris water maze (MWM). (A) Escape latency to find the submerged platform over 12 trials for three consecutive days was assessed through two‐way RM‐ANOVA. (B) One‐way ANOVA analysis of calculated area‐under‐the‐curve (AUC) for the escape latencies for 12 trials over 3 days during the acquisition phase (four trials per day). (C) Distance traveled to find the submerged platform over 12 trials was assessed through Two‐way RM‐ANOVA. (D) One‐way ANOVA analysis of calculated AUC for the distance traveled to platform for 12 trials over 3 days during the acquisition phase. (E) Time spent in the target quadrant searching for platform during day 4 of the test (probe phase). (F) Number of crossings over the previously hidden platform area in probe phase. (G) Escape latency in probe phase. (H) Overall swimming distance during 60 sec of probe phase. (I) Overall swimming speed on day four of the MWM test. Data are represented in the form of mean ± SEM (*n* = 6/group). *p < 0.05, **p < 0.01, ***p < 0.001, ****p < 0.0001 vs. control; ^*p* < 0.05, ^^*p* < 0.01, ^^^^*p* < 0.0001, vs. AM140 + Aßo. ####*p* < 0.0001 vs. AβO. (Ctrl, control; AßO, oligomer amyloid β; AM140, Anti‐microRNA‐140‐5p; AUC, area‐under‐the‐curve)

To verify the effect of miR‐140‐5p inhibitor on learning tasks and hippocampus‐dependent behavioral memory abilities, we examined the hippocampal‐dependent spatial memory of rats using the MWM task on days 18–21 after surgery. All the experimental groups, regardless of the treatments, indicated progressively reduced escape latencies and swimming distance during the 3 days of cue training (Figure 2A,C). Although, the escape latencies and traveled distance reported for the AßO group did not have a rapid or robust descending pattern as has been observed for other groups. Two‐way RM‐ANOVA revealed significant effects of both time factor (indicating learning performance) (F _[11, 308]_ = 15.68, *p* < 0.0001) and treatment (F _(3, 28)_ = 12.05, *p* < 0.0001) (Figure 2A). For further comparison and simplifying the statistical analysis in the multiple measurements,^37^ the areas under the learning curves (AUCs) for 12 trials over 3 days during the acquisition phase (four trials per day) were calculated for each animal (F _(3, 27)_ = 13.39, *p* < 0.0001) (Figure 2B). AUC calculation for the escape latencies was significantly increased in the AßO group compared with healthy control rats (*p* < 0.001), while it was substantially reduced in rats received both AM140 and AßO (*p* < 0.0001 vs. AßO group). As indicated in Figure 2C, learning curve was also analyzed by path length, which also showed significant effects of both time (indicating learning performance) (F _[11, 330]_ = 15.17, *p* < 0.0001) and treatment (F _(3, 30)_ = 17.91, *p* < 0.0001) through RM‐ANOVA (Figure 2C). AUC analysis of the parameter ‘path length’ was performed through one‐way ANOVA (F _(3, 27)_ = 25.95, *p* < 0.0001) and revealed that Aßo rats traveled longer distances relative to controls (*p* < 0.0001), whereas AM140 significantly limited the distances traveled by AßO received rats during 3 days of the acquisition phase (*p* < 0.0001) (Figure 2D). In the probe trial, AßO‐treated group significantly spent less time in the target quadrant searching for platform compared with controls (*p* < 0.05), however, rats with co‐injection of AM140 with AßO showed an increase in the duration F _(3, 27)_ = 3.399, *p* = 0.0320) (Figure 2E). In the probe phase, there was no significant difference in the number of crossings over the previously hidden platform area among the four groups (F _(3, 28)_ = 1.199, *p* = 0.3281) (Figure 2F). The parameter escape latency in the probe phase was significantly delayed in the Aßo group (*p* = 0.0141), and AM140 treatment did not make any significant difference in escape latency compared with AD‐like rats (*p* = 0.9535) (F _(3, 31)_ = 7.476, *p* = 0.0007) (Figure 2G). Toward one‐way ANOVA statistical analysis, no significant alteration in overall swimming distance (F _(3, 28)_ = 1.450, *p* = 0.2495) and speed (F _(3, 28)_ = 2.082, *p* = 0.1252) were observed on day four of the MWM test (Figure 2H,I, respectively).

### 3.2 mir‐140‐5p inhibitor decreased the mir‐140 expression levels in the hippocampus on day 7 post‐injection

**FIGURE 3:**
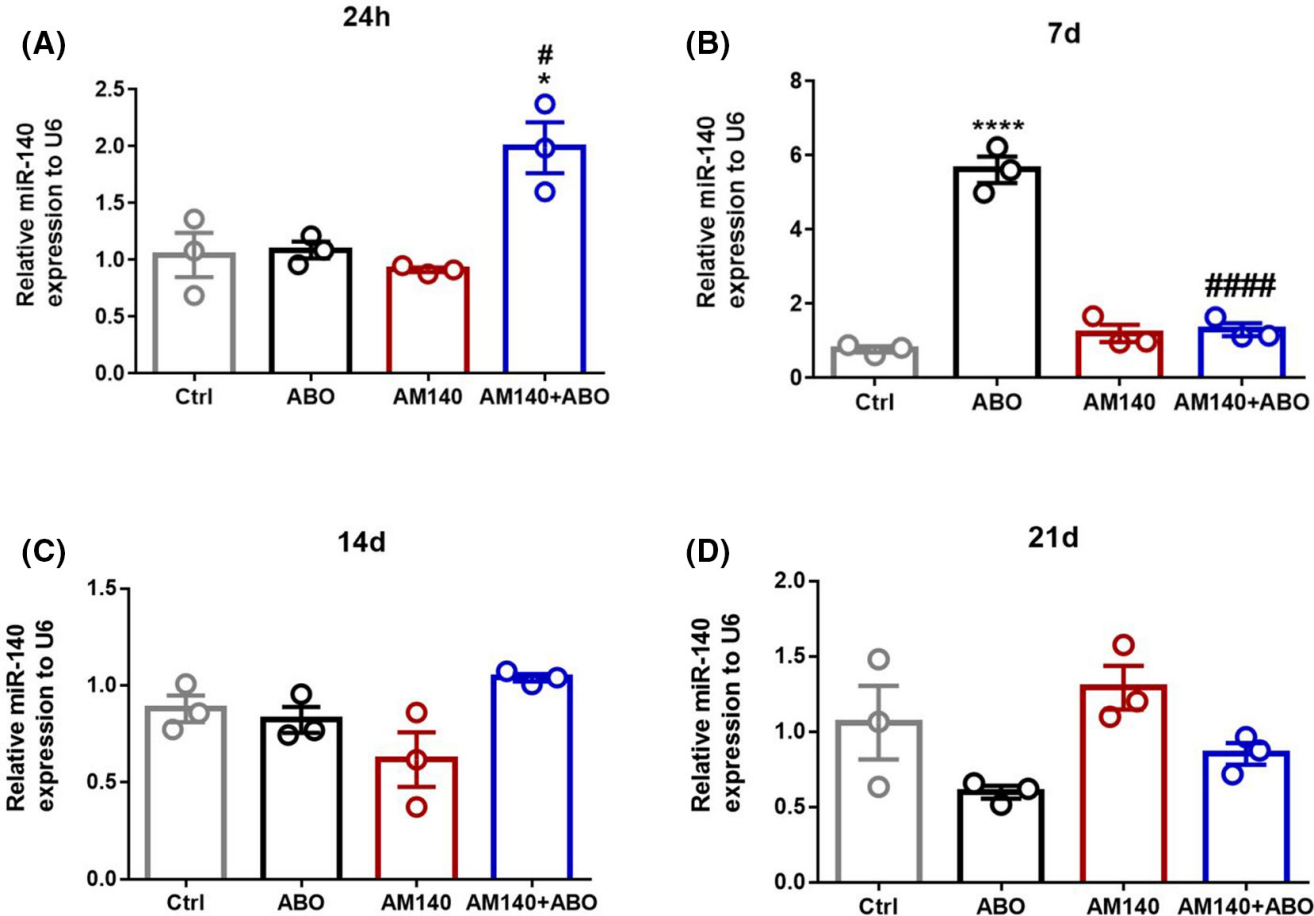
Mir‐140 expression on day 1 (A), 7 (B), 14 (C), and 21 (D) following ABO and AM140 administration was tested by quantitative PCR (qPCR; *n* = 3/group). Data are shown as mean ± SEM values. A one‐way ANOVA test was used for the comparison of multiple groups. Statistical significance between groups is defined as *p < 0.05, **** *p* < 0.0001 vs. control and ####*p* < 0.0001 vs. AßO (Ctrl: Control group, AßO: Amyloid Beta Oligomer, AM140: AntimiR‐140‐5p)

We assessed the mir‐140 expression at different times (24 h, 7, 14, and 21 days) after stereotactic injections using RT‐qPCR. 24 h post‐surgery, a marked increased expression was observed only in the AM+Aβo group compared with control and Aβo groups (F _(3, 8)_ = 10.34, *p* = 0.0040) (Figure 3A). On the next time point, day 7, a single dose i.h. injection of AßO resulted in a significant increase in mir‐140 expression in the hippocampus of Aβo rats in comparison with the vehicle‐treated group (*p* < 0.0001). Treatment of rats with mir‐140‐5p inhibitor considerably reduced the mir‐140 expression levels on day 7 in AßO + AM140 group vs. healthy rats (F _(3, 8)_ = 94.12, *p* < 0.0001) (Figure 3B). However, in the later time points of day 14 (F _(3, 8)_ = 4.156, *p* = 0.0476) (Figure 3C) and 21 (F _(3, 8)_ = 3.991, *p* = 0.0522) (Figure 3D) no significant differences were found in the levels of mir‐140 expression among the four experimental groups.

### 3.3 mir‐140‐5p inhibition improved Pin1 and ADAM10 mRNA expression levels in AßO injected rats

**FIGURE 4:**
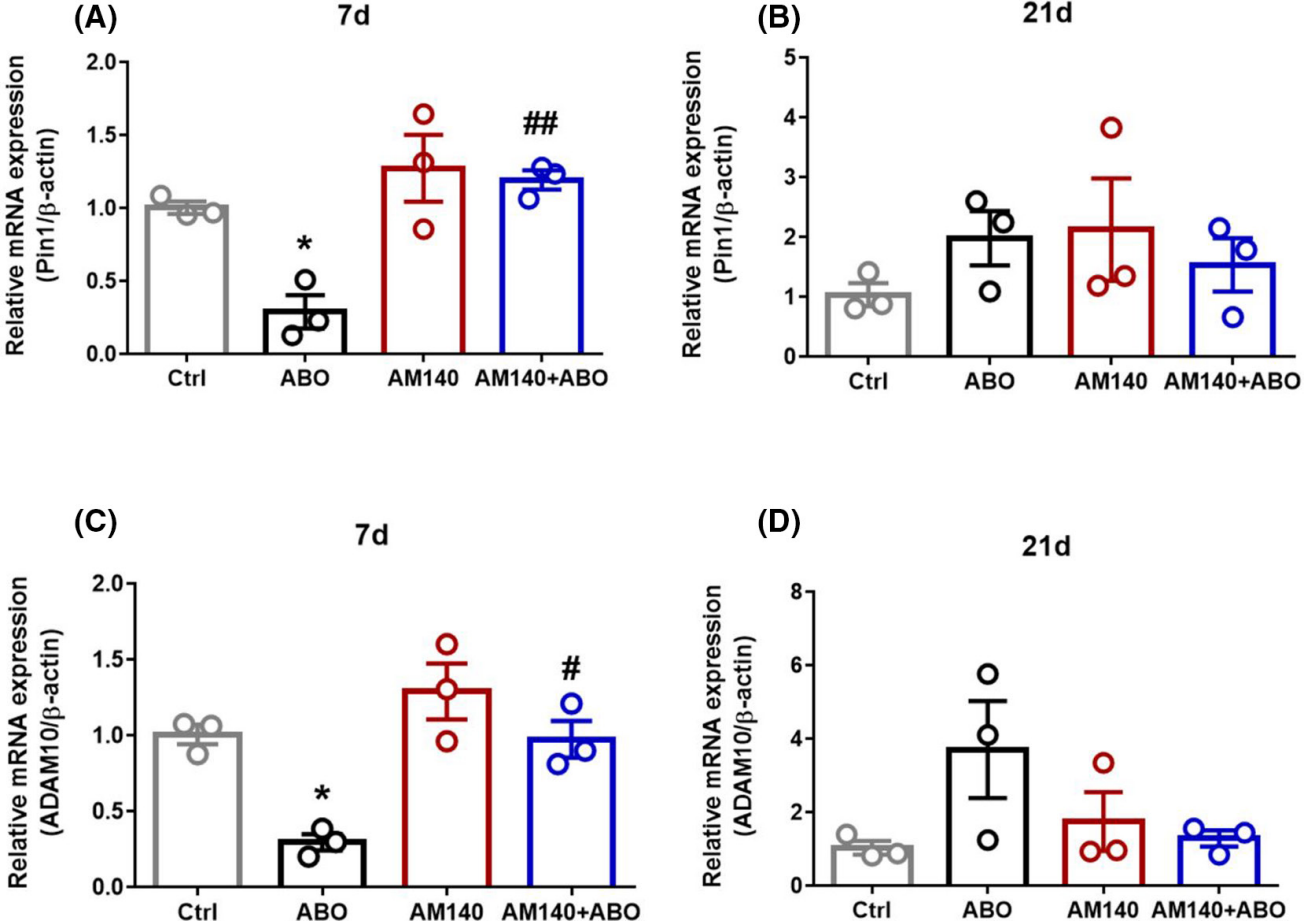
Pin1 and ADAM10 mRNA expression on days 7 (A,C) and 21 (B,D) following ABO and AM140 administration were tested by quantitative PCR (qPCR; *n* = 3/group). Data are shown as mean ± SEM values. A one‐way ANOVA test was used for comparison of multiple groups (Tukey’s post hoc). Statistical significance is defined as *p < 0.05 compared with control group, # *p* < 0.05 and ## *p* < 0.01 compared with ABO group. (Ctrl: Control group, ABO: Amyloid Beta Oligomer, AM140: Antimir‐140)

We next analyzed whether or not the i.h. injection of AßO would result in a lower mRNA expression of Pin1 and ADAM10 (the main targets of mir‐140‐5p) as well as to investigate the effects of the mir‐140‐5p inhibitor on the mRNA expression levels may occur, and for how long it might be maintained in the hippocampus. Statistical analysis revealed that there was a significant difference in Pin1 ((F _(3, 8)_ = 11.18, *p* = 0.0031) (Figure 4A)) and ADAM10 ((F _(3, 8)_ = 12.81, *p* = 0.0020) (Figure 4C) mRNA expression between groups on day 7 post‐surgery. A single exposure of AβO was associated with a marked decrease in mRNA expression of Pin1 (*p* < 0.05) and ADAM10 (*p* < 0.05) compared with healthy controls on day 7 post‐surgery. Mir‐140‐5p inhibitor alone did not make any significant change in both mRNA levels of Pin1 and ADAM10 in healthy rats, however, its administration following the AβO injection significantly increased the expression level of Pin1 (*p* < 0.01), and ADAM10 (*p* < 0.05) in comparison with AßO injected rats. As shown in Figure 4B, on day 21 of the study, Pin1 expression level did not differ significantly between groups (F _(3, 8)_ = 0.8194, *p* = 0.5187), although a statistically insignificant elevated level of ADAM10 was detected in AßO group (F _(3, 8)_ = 2.382, *p* = 0.1452) (Figure 4D).

### 3.4 mir‐140‐5p inhibitor attenuated AßO induced mRNA expression of IL‐1ß and TNF‐α in the hippocampus

**FIGURE 5:**
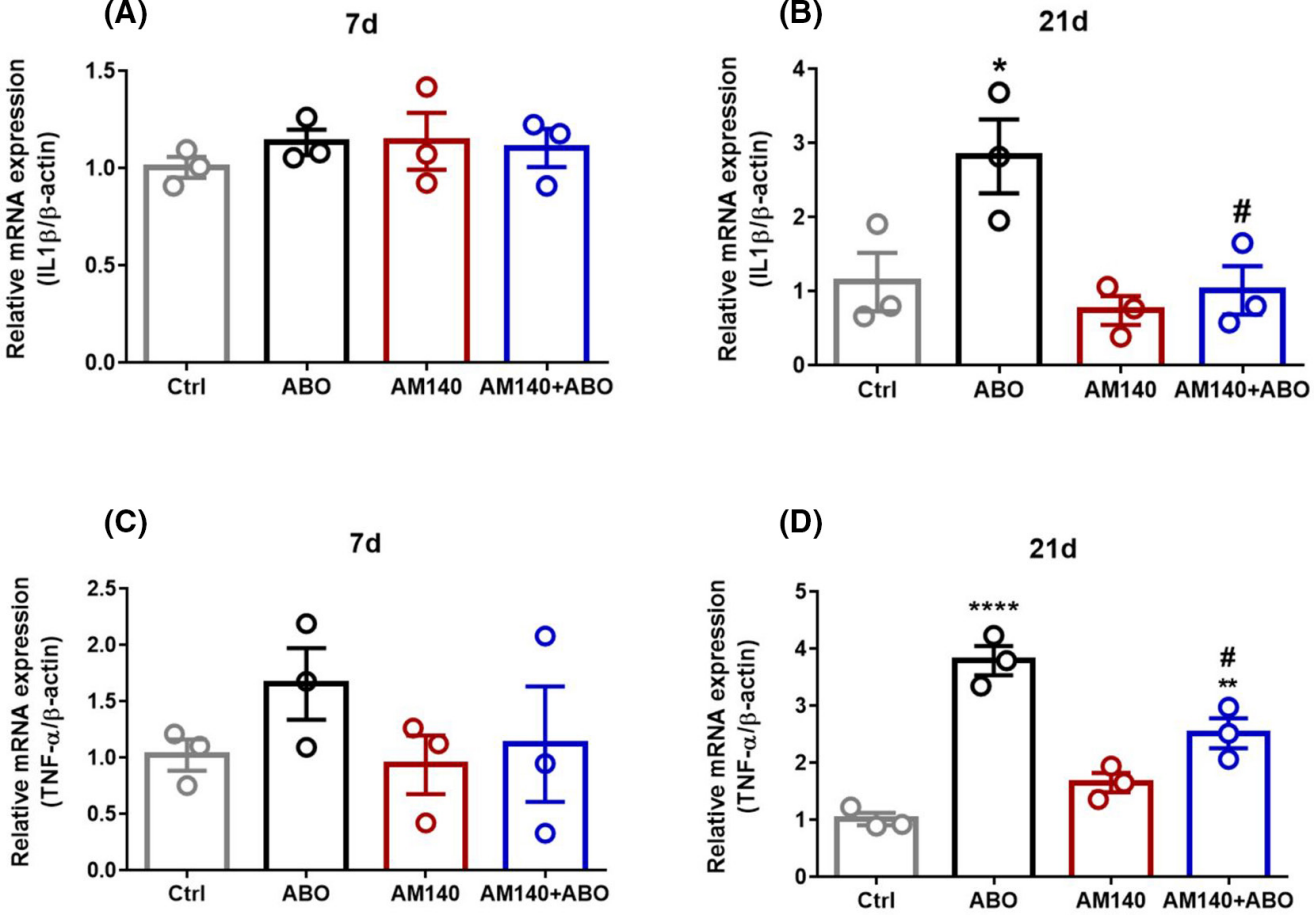
IL‐1ß and TNF‐α mRNA expression on day 7 (A,C) and 21 (B,D) following ABO and AM140 administration was tested by quantitative PCR (qPCR; *n* = 3/group). Data are shown as mean ± SEM values. A one‐way ANOVA test was used for comparison of multiple groups (Tukey’s post hoc). Statistical significance is defined as * *p* < 0.05, ** *p* < 0.01, and **** *p* < 0.0001 compared with control group, # *p* < 0.05 compared with AßO group (Ctrl: Control group, AßO: Amyloid Beta Oligomer, AM140: Antimir‐140)

In order to obtain further evidence on the role of miR‐140‐5p in the AßO induced learning damage and memory decline, possible alterations in mRNA expression of the key inflammatory mediators (IL‐1ß and TNF‐α) were also evaluated in hippocampus samples on day 7 and 21 post‐surgery. No remarkable difference was found in fold change values of IL‐1ß (F _(3, 8)_ = 0.4047, *p* = 0.7538) (Figure 5A) and TNF‐α (F _(3, 8)_ = 0.9260, *p* = 0.4712) (Figure 5C) between groups on day 7. Nevertheless, on day 21, one‐way ANOVA indicated a significant effect of treatment on IL‐1ß and TNF‐α mRNA expression ((F _(3, 8)_ = 6.504, *p* = 0.0154) and (F _(3, 8)_ = 33.00, *p* < 0.0001), respectively). AßO administration markedly enhanced the mRNA expression of IL‐1ß (*p* < 0.05) (Figure 5B) and TNF‐α (*p* < 0.0001) (Figure 5D) compared with healthy control group. Interestingly, rats treated with miR‐140‐5p inhibitor after AßO administration showed a significant decrease in IL‐1ß (*p* < 0.05) and TNF‐α (*p* < 0.05) versus AßO group.

### 3.5 Significant protective changes were observed in most of the Pin1 target proteins following miR‐140‐5p inhibitor administration in AßO received rats

**FIGURE 6:**
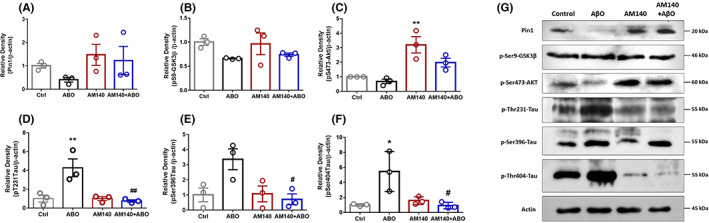
Pin1 (A), pS9‐GSK3ß (B), pS473AKT (C), pT231tau (D), pS396tau (E), and pS404tau (F) protein expression on day 21 following AβO and AM140 administration was tested by western blot analysis method (*n* = 3/group). (G) Representative Western blot analysis from the hippocampus of rats showing the above‐mentioned protein expression levels. Data are shown as mean ± SEM values. A one‐way ANOVA test was used for the comparison of multiple groups (Tukey’s post hoc). Statistical significance is defined as * *p* < 0.05, and ** *p* < 0.01 compared with control group, # *p* < 0.05 and ## *p* < 0.01 compared with AßO group (Ctrl: Control group, AßO: Amyloid Beta Oligomer, AM140: Antimir‐140)

To determine whether AM140 might ameliorate the spatial learning and memory on day 21 through upregulation of Pin1, western blot analysis was performed to measure the protein expression level of Pin1. Although the AßO group seemed to display a lower Pin1 protein expression, and increased levels of Pin1 were also observed in both AM140 and AM140‐treated AßO groups, one‐way ANOVA analysis did not show any significant changes in Pin1 expression levels among the four groups (F _(3, 8)_ = 1.424, *p* = 0.3056) (Figure 6A). The expression levels of phosphorylated Ser473‐Akt (pS473‐Akt) and phosphorylated Ser9‐GSK3ß (pS9‐ GSK3ß) were also assessed to evaluate changes in the key components of insulin signaling pathways. As indicated in Figure 6B, there is no significant difference between the experimental groups in pS9‐GSK3ß protein expression on day 21 of the study (F _(3, 8)_ = 1.980, *p* = 0.1956). However, one‐way ANOVA demonstrated a marked effect of treatment on pS473‐Akt protein expression on day 21 between groups (F _(3, 8)_ = 11.96, *p* = 0.0025). Although pS473‐Akt expression level had no statistically significant difference in the AßO group compared with control, AM140 administration caused a significant increase in the pS473‐Akt expression in healthy rats (*p* < 0.01) and was also associated with an elevated level of the protein in AßO rats (*p* = 0.0892) (Figure 6C).

In subsequent studies, phosphorylated tau proteins, including Thr231 (pT231), Ser396 (pS396), and Ser404 (pS404) were evaluated to determine whether the treatment with AM140 influenced the phosphorylation status of tau in AD‐like conditions. The results obtained 21 days post‐surgery indicated a significant difference in pT231 tau between groups (F _(3, 8)_ = 10.27, *p* = 0.0041). As depicted in Figure 6D, the AßO group was associated with a significant increase in pT231 tau protein expression compared with controls (*p* < 0.01). AM treatment caused a markedly lower level of pT231 tau in rats after exposure to AßO (*p* < 0.01). There was also a statistically significant difference between groups in the expression of tau phosphorylated in the Ser396 region (F _(3, 8)_ = 5.540, *p* = 0.0236) (Figure 6E). A statistically significantly lower pS396 tau expression was detected in the AM140 + AßO group compared with AßO rats (*p* < 0.01). Figure 6F demonstrates a significant difference between groups in pT404 tau expression (F _(3, 8)_ = 7.477, *p* = 0.0104). A significantly elevated protein level of pT404 tau was found in rats that received only AßO (*p* < 0.05) versus healthy rats, and AM140 administration attenuated its expression in the hippocampus of AM140+ AßO rats in comparison with the AßO group (*p* < 0.05). All the original, uncropped blot images were provided in the Supplementary file S1.

### 3.6 Observation of nissl‐stained sections

**FIGURE 7:**
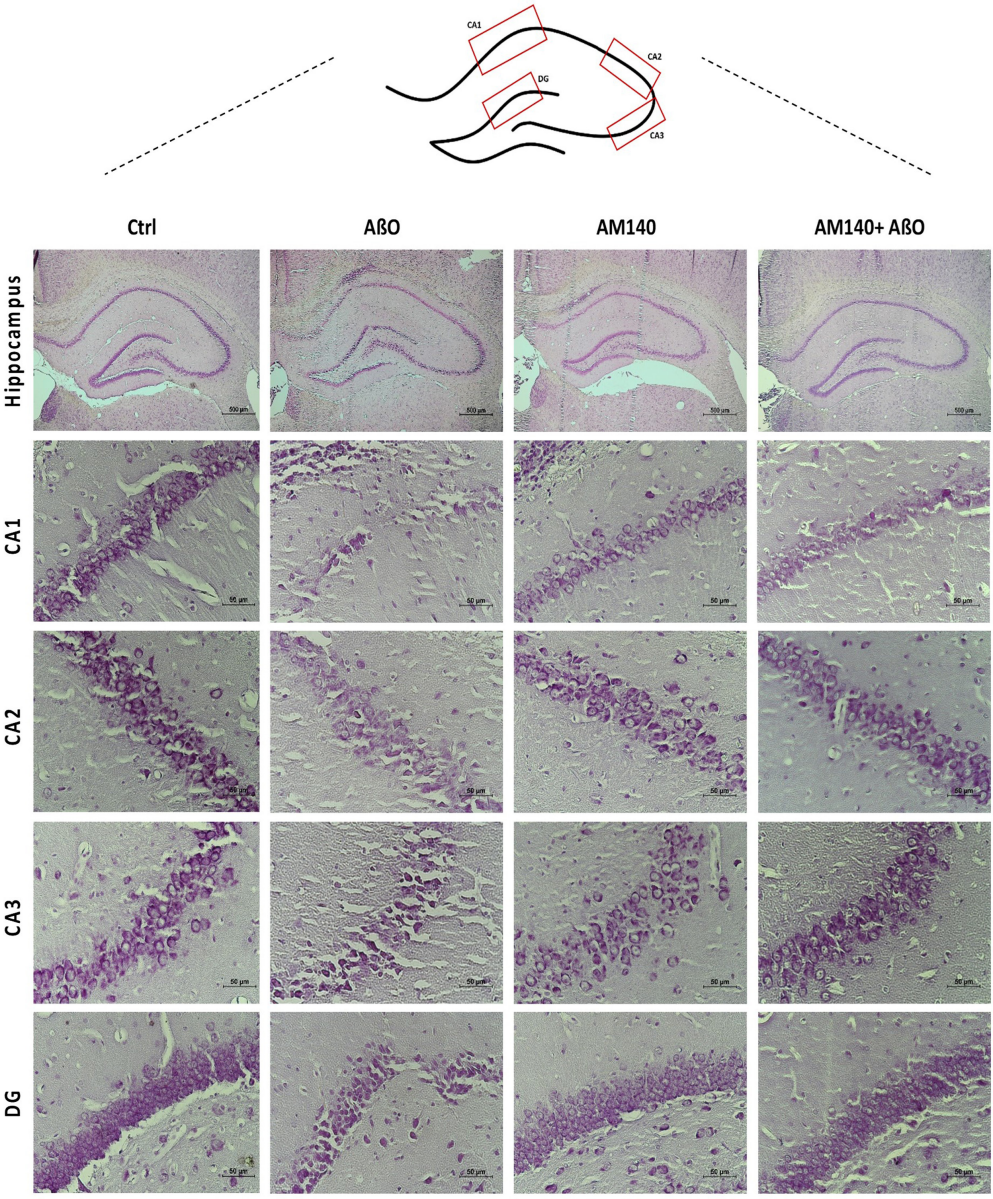
Hippocampal morphological changes following AD induction and the effect of AM140 on the histopathology of different areas of the hippocampus. Nissl staining was applied to identify Nissl bodies and the extent of neuronal damage in the hippocampus (×4), CA1, CA2, CA3, and DG region subfields (×40). Scale bar =500 μm (general) and 50 μm (regions). Ctrl, control; Aβo, oligomer amyloid β; AM140, Anti‐microRNA‐140‐5p, DG, dentate gyrus

The whole‐brain sections collected on day 21 were stained with cresyl violet to analyze the morphology of the hippocampus in the different groups (Figure 7). Nissl staining presented that control and AM140‐treated rats had normal hippocampal tissues, and cells were arranged tidily and stained uniformly in any of the tested brain regions. However, in the AßO group, nissl‐stained dark neurons with abnormal morphologies of massive shrunken were identified in a large number in the CA1, the CA3, and the dentate gyrus regions. Considerable improvement was observed in the AM140+ AßO group, and the number of dead cells showed a marked decrease versus the AßO group.

## 4 DISCUSSION

In the present study, we addressed the question of whether miR‐140‐5p inhibition in rat hippocampus can be useful to target the main pathological processes of AD, namely, impaired cognitive function, neuroinflammation, hyperphosphorylation of tau in the brain, and defective brain insulin signaling. To our knowledge, up to now, no convincing data is showing that this strategy may buffer against AD cascades in vivo. Thus, we examined the preventive effect of AM140 against AβO and its pathological cascades. Previous reports have described soluble Aβ oligomers as the primary toxic agent that mediate early memory dysfunction and synaptic degeneration in AD rather than insoluble amyloid plaques.^38^

The primary conclusion of the current study is that i.h. injection of AM140 inhibitor results in an improvement of learning and memory deficits in AβO‐induced AD‐like conditions, which was examined by MWM test on day 21 following the agents' administration. Treatment of AD‐like animals with AM140 was associated with a significant improvement in the parameters studied in the learning phase, including escape latency and the path length. In the probe phase, positive changes were also observed in the time taken to reach the original platform position, although similar changes were not observed in other parameters for memory evaluation.

A number of recent studies have found miR‐140‐5p upregulation to be involved in AD pathology.^19^, ^39^ Furthermore, in a study by Gullett et al., miR‐140‐5p has been introduced as one of the top‐ranked predictors of age‐related cognitive decline.^22^ Based on bioinformatics and biological proofs, Pin1 and ADAM10 could be the most important targets for miR‐140‐5p in AD‐related conditions^11^, ^19^ and the role of miR‐140‐5p as a negative post‐transcription regulator of pin1 has been indicated in recent publications (Yan et al., 2017; Heidari et al., 2018). Consistently, the loss of peptidyl‐prolyl cis/trans isomerase Pin1 activity has revealed its indispensable role in the disruption of synaptic plasticity,^40^ tau‐related pathology,^41^ and promoting amyloidogenic APP processing.^42^ It has been found that hippocampal Pin1 protein expression is significantly lower in AD brains than in age‐matched normal controls.

In this work, we presented evidence that AßO, as the leading neurotoxin in the AD progression, can increase miR‐140‐5p expression, leading to a significant decrease in Pin1 and ADAM10 mRNA levels, only on day 7 following the i.h. injection. Furthermore, AM140 treatment effectively suppressed the miRNA‐140 elevated level, which was simultaneously associated with improvements in Pin1 and ADAM10 mRNA expression levels. Nevertheless, the cause of the ambiguous expression pattern of miR‐140 on day one after the surgery and its upregulation only in AM140‐treated AD‐like rats remained questioned and more studies should be done on how the level of the miRNA changes in shorter time intervals. On the other hand, Pin1 expression on protein level was only analyzed on day 21 after the initial insult, which was not associated with a clear significant difference between treated and untreated animals. It should come as no surprise that this was the major limitation of this study and analysis of Pin1 protein level at a time‐point close to day 7 could therefore be more helpful.

In the next part of the study, the association of miR‐140‐5p downregulation with some of the main neuropathological hallmarks of AD was examined. It is well known that neuroinflammation plays an important role in cognitive impairment and AD development. It is reported that AßO stimulates neuroinflammation through the direct activation of microglia, which in turn lead to the release of inflammatory cytokines, and ultimately to neuronal cell loss.^43^, ^44^ Similarly, here, we found that i.h. injection of Aß 1–42 oligomer significantly increased the levels of IL‐1ß, and TNF‐α mRNA expression in hippocampal tissue on day 21 post‐injection, which were ameliorated following AM140 treatment. Therefore, repressing the AßO‐triggered neuroinflammation could be one of the possible protective mechanisms for AM140 in cognitive decline.

The most apparent consequence of AM140 administration in AD‐like rats over 21 days was ameliorating the phosphorylation levels of tau at three studied sites (Thr231, Ser396, and 404) induced by AßO injection. Tau phosphorylation at the Thr231 region is thought to be an early crucial event prior to NFT formation.^45^ pThr231 at cis conformation, but not trans, is less vulnerable to dephosphorylation/degradation and strongly correlates with tau accumulation, tangle formation, and thus nerve degeneration.^3^, ^46^, ^47^, ^48^ It has been also introduced as a potential predictor for the conversion of human mild cognitive impairment to AD.^49^ Ser396 and Ser404 are also phosphoepitopes found in hyperphosphorylated tau in the AD hippocampus, particularly at early stages.^50^ Notably, Pin1 has been indicated to catalyze a local cis‐trans conformational change of pSer/Thr‐Pro motif in tau which directly restores tau's function, indirectly accelerates its dephosphorylation by protein phosphatase 2A, and inhibits the access of GSK3β for further phosphorylation.^51^, ^52^

The indispensable role of aberrant neuronal Insulin signaling in AD brains has been confirmed through a huge number of studies.^53^, ^54^, ^55^ On the other side, there is some evidence suggesting that insulin resistance is associated with miR‐140‐5p upregulation.^56^ Consistently, previous studies have reported that agents enhancing Pin1 activity or expression may serve as a potential treatment for insulin resistance states, such as diabetes mellitus Type 2.^57^ Previously, Wang et al. showed that miR‐140‐5p could inhibit the PI3K/AKT signaling pathway and its expression is negatively correlated with the expression of pSer473Akt and p‐PI3K in osteoarthritis tissues.^58^ Here, although AßO made no remarkable changes in the key elements of the signaling pathway, including pSer473Akt and pSer9GSK3β compared with control on day 21, employing AM140 alone could significantly upregulate pSer473Akt in hippocampal samples, which may be due to the suppression of the intrinsic effects of miR‐140‐5p in healthy brains. However, the inhibitor did not induce any significant change in the component in AD‐like condition.

Our study only provides preliminary but informative evidence for our underlying hypothesis regarding the therapeutic potential of miR‐140‐5p modulation in AD patients and suffers from a number of limitations. Here, we conducted multi‐time‐point surveys to evaluate the effects of miR‐140‐5p inhibitor on AD's main pathological hallmarks. However, the exact prediction of the time‐course pharmacological properties of anti‐miRs is often complicated, since their pharmacological activities have been found to be related to the sum of pharmacological effects over time that leads to alterations in the disease phenotype.^59^ Our work confirms the effects of miR‐140‐5p inhibition in improving cognitive performance in preclinical settings in MWM task, a finding that seems it would benefit from replication in a larger sample size. As in the present study, no significant difference in Pin1 protein expression was found in treatment effect among groups, it would be extremely difficult to link the observed protective effects of the miR‐140‐5p inhibitor to enhanced levels of Pin1 expression. Assessing the ADAM10 expression at the protein level can also be helpful to better judge the mechanism of action of the miRNA inhibitor. Moreover, further investigations will be needed to study the relative effects of miR‐140‐5p inhibition on AD pathology in species that have miRNA‐mRNA interaction networks more similar to humans. A well‐designed in vivo experiment using animal models with the full complexity of the human pathology is warranted to comprehensively understand the protective role of the AM140 in AD treatment. Besides, using miRNA inhibitors with more reliable chemical modifications such as conjugation to a cholesterol group to enhance the cellular uptake may help to overcome pharmacokinetic challenges.^60^ Enhanced methods of targeted brain delivery should also be considered, as it has been recognized as a major barrier to using antisense oligonucleotides for chronic neurodegenerative diseases in the clinic.^61^ Such studies may help toward examining the prospects for therapeutically miR‐140‐5p manipulation to improve cognitive performance in an AD‐like context.

## 5 CONCLUSION

This study exhibits a crucial role for miR‐140‐5p in the key events of oligomeric amyloid‐beta induced neurotoxicity, which may serve as a potential therapeutic target for AD. Our preclinical study also provided evidence that the local silencing of miR‐140‐5p within the hippocampus may attenuate memory impairment and prevents disease progression in patients suffering from AD.

## AUTHOR CONTRIBUTIONS

**Pariya Khodabakhsh:** investigation, acquisition, analysis, and interpretation of data, writing—original draft. **Maryam Bazrgar:** investigation, acquisition, and interpretation of data. **Fatemeh** **Mohagheghi:** conceptualization and supervision. **Siavash Parvardeh and Abolhassan** **Ahmadiani:** conceptualization, methodology, supervision, and funding acquisition. All authors revised and approved the final version of the manuscript. All persons who meet authorship criteria are listed as authors, and all authors certify that they have participated sufficiently in the work to take public responsibility for the content, including participation in the concept, design, analysis, writing, or revision of the manuscript. Furthermore, each author certifies that this material or similar material has not been and will not be submitted to or published in any other publication before its appearance in the CNS Neuroscience & Therapeutics journal.

## FUNDING INFORMATION

This study was funded by Research Affairs (Grant No. 13.42) of Shahid Beheshti University of Medical Sciences, Tehran, Iran.

## CONFLICT OF INTEREST

The authors declare that the research was conducted in the absence of any commercial or financial relationships that could be construed as a potential conflict of interest.

## Supporting information


Appendix S1

## Data Availability

The raw data supporting the conclusions of this article will be made available by the authors upon reasonable request.
