# Supplementary material for: MicroRNA‐140‐5p inhibitor attenuates memory impairment induced by amyloid‐ß oligomer in vivo possibly through Pin1 regulation
Source: CNS Neurosci Ther. 2022 Oct 2;29(1):91–103. doi: 10.1111/cns.13980 (PMC9804077; doi:10.1111/cns.13980)
Supplement: Supplementary file 1 — Appendix S1 [file CNS-29-91-s001.pdf]

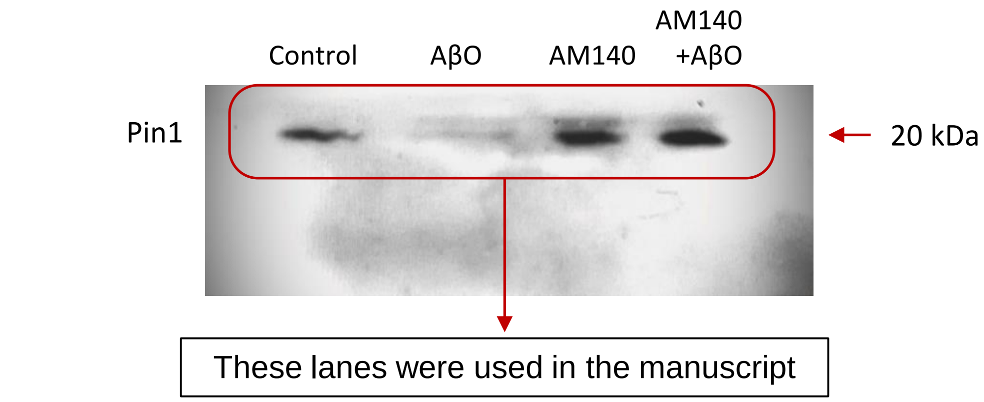
Control
AβO
AM140
AM140 +AβO
Pin1
20 kDa
These lanes were used in the manuscript

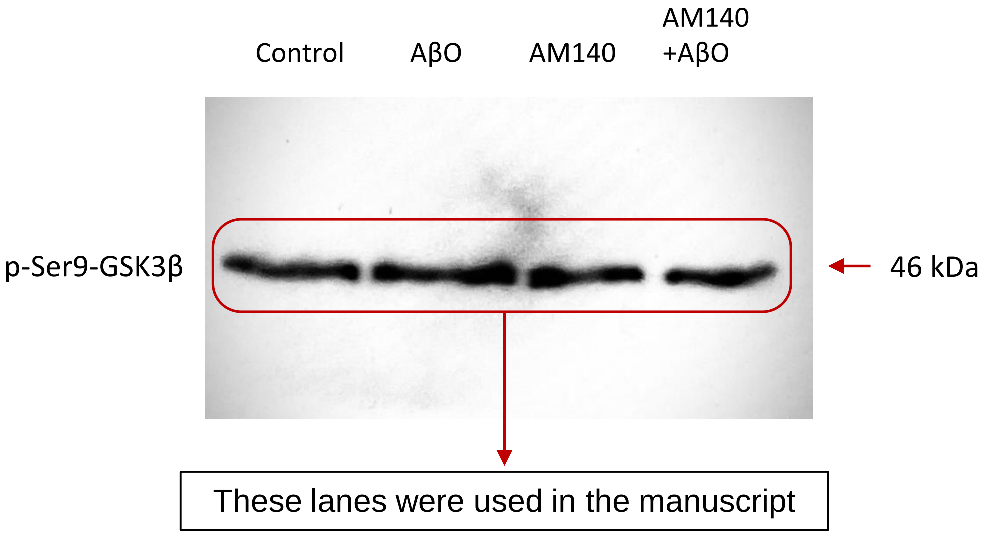
Control
AβO
AM140
AM140 +AβO
p-Ser9-GSK3β
46 kDa
These lanes were used in the manuscript

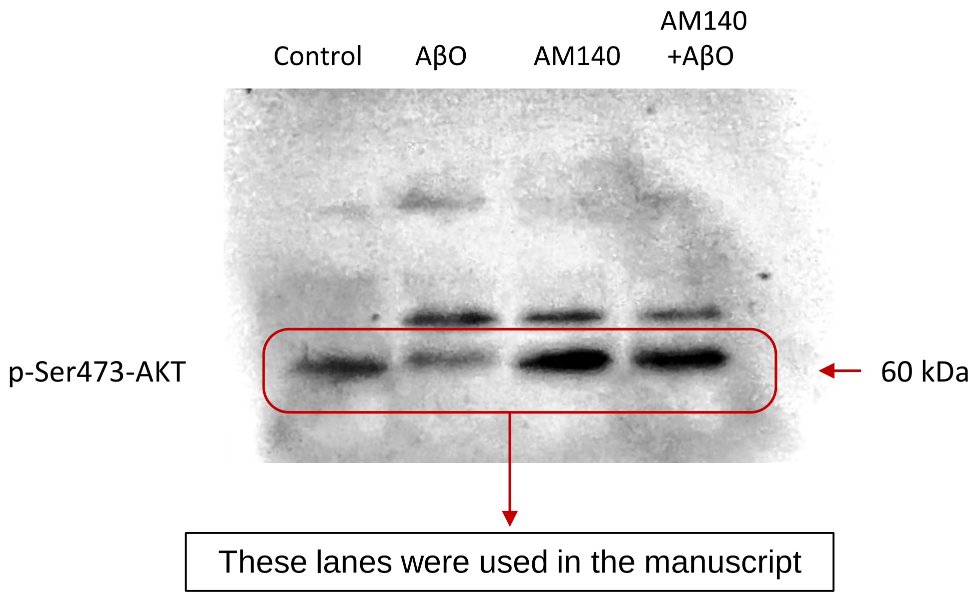
Control
AβO
AM140
AM140
+AβO
p-Ser473-AKT
60 kDa
These lanes were used in the manuscript

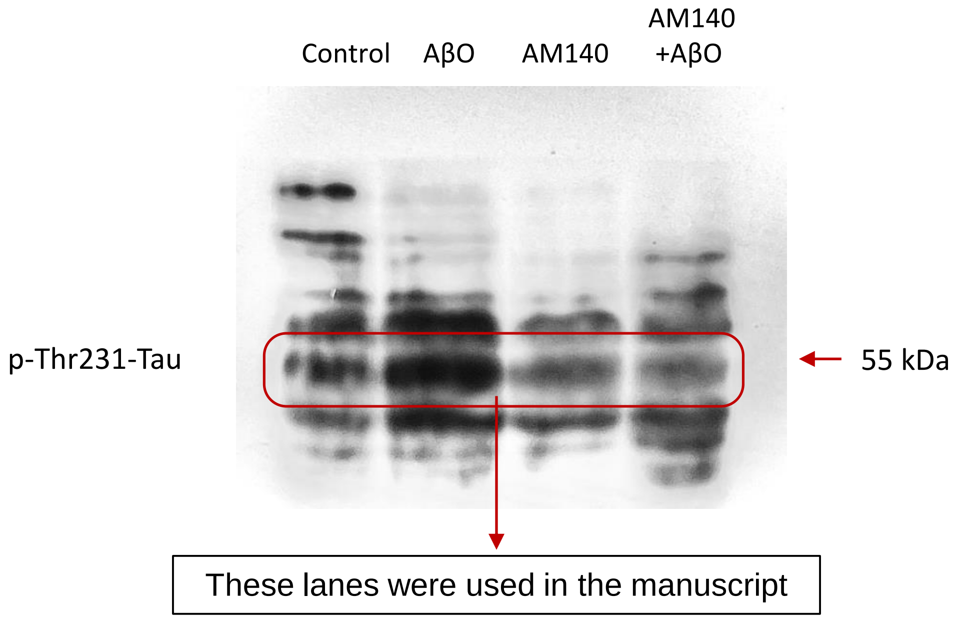
Control
AβO
AM140
AM140
+AβO
p-Thr231-Tau
55 kDa
These lanes were used in the manuscript

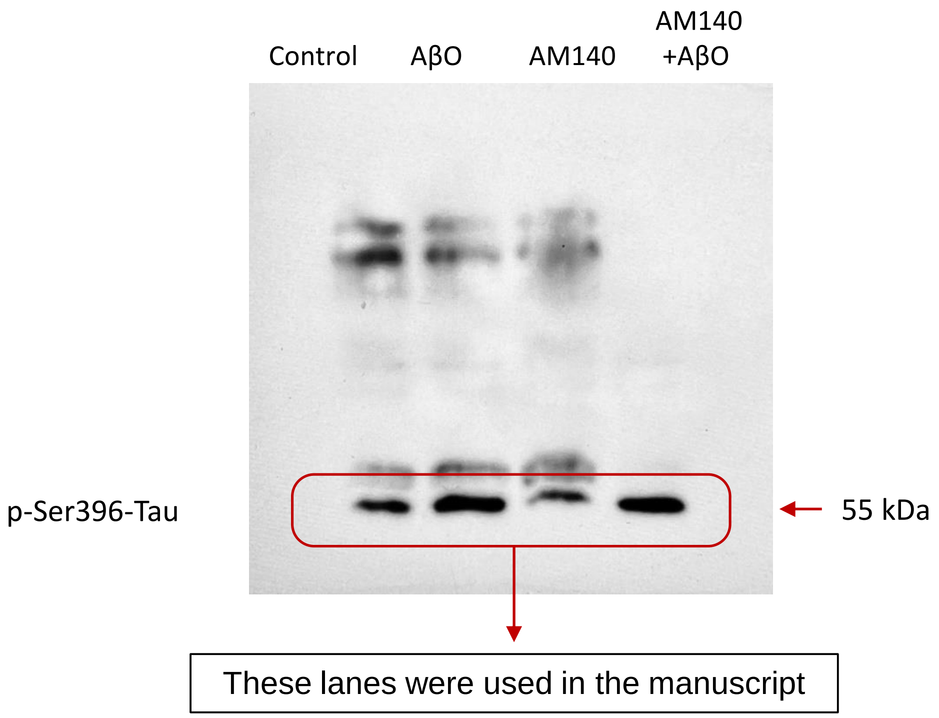
Control
AβO
AM140
AM140
+AβO
p-Ser396-Tau
55 kDa
These lanes were used in the manuscript

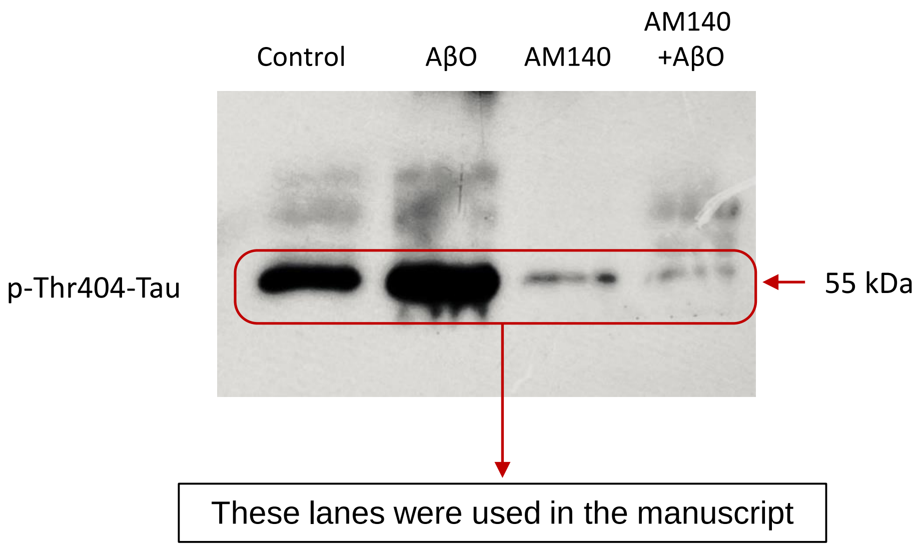
Control
AβO
AM140
AM140
+AβO
p-Thr404-Tau
55 kDa
These lanes were used in the manuscript

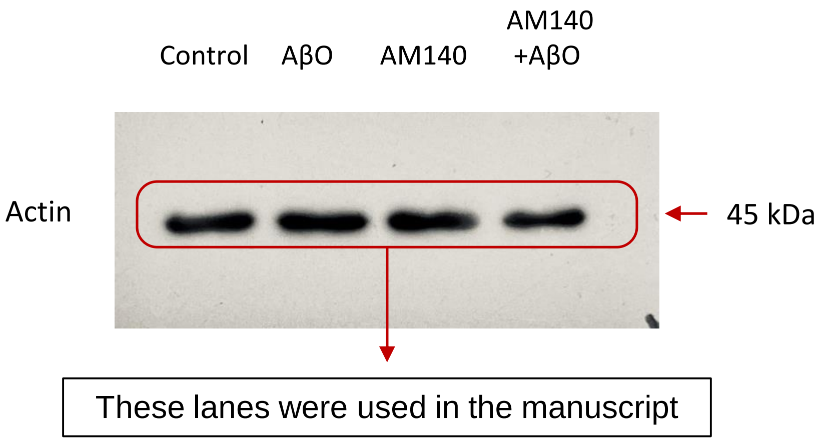
Control
AβO
AM140
AM140 +AβO
Actin
45 kDa
These lanes were used in the manuscript
